# Experimental Glomerular Endothelial Injury *In Vivo*

**DOI:** 10.1371/journal.pone.0078244

**Published:** 2013-10-15

**Authors:** George Haddad, Lin Fu Zhu, David C. Rayner, Allan G. Murray

**Affiliations:** 1 Department of Medicine, University of Alberta, Edmonton, Alberta, Canada; 2 Department of Surgery, University of Alberta, Edmonton, Alberta, Canada; 3 Department of Pathology and Laboratory Medicine, University of Alberta, Edmonton, Alberta, Canada; UCL Institute of Child Health, United Kingdom

## Abstract

The microvascular endothelium of the kidney glomerulus is injured in Shiga-like toxigenic bacterial infection, genetic or acquired loss of complement regulatory protein function, and allo-immune responses of solid-organ or bone marrow transplantation. Existing models of diseases with glomerular endothelial cell (EC) injury, collectively grouped as thrombotic microangiopathies, are problematic, impeding investigation of the mechanisms of microvascular defense and repair. To develop a model of glomerular endothelial injury in the mouse, we conjugated the *M. oreades* lectin to the cytotoxin, saporin, (LS) to selectively injure the glomerular endothelium. Injury of the microvasculature was evaluated by light, immunofluorescence, and electron microscopy, and by quantitative RT-PCR of cell-type specific transcripts. Renal function was evaluated by quantitation of serum creatinine. The toxin conjugate induced apoptosis of microvascular ECs *in vitro*, and subtle histologic features of thrombotic microangiopathy *in vivo* that were enhanced by co-injection of 50 μg/kg LPS. Among LS/LPS-treated animals, loss of glomerular EC staining correlated with decreased expression of EC-specific transcripts, and impaired kidney function. Selective injury of the glomerular microvasculature with LS toxin conjugate and LPS elicits histologic features of thrombotic microangiopathy and acute kidney failure.

## Introduction

The vascular endothelium is the principal target of injury in a group of disorders collectively termed thrombotic microangiopathies (TMA). TMA is initiated by diverse processes including uncontrolled complement protein activity caused by inherited or acquired defects in complement regulatory proteins, cytotoxic drugs, or immune responses to allogeneic endothelium in the context of allogeneic bone marrow or solid organ transplantation. The most common cause of TMA is Shiga-toxigenic (Stx) *E coli* infection. TMA injury occurred in about 25% of infected cases to contribute to the high death rate observed in the recent German epidemic [1,2], and may be associated with chronic renal dysfunction among survivors [3,4]. Glomerular endothelial injury associated with the atypical hemolytic-uremic syndrome frequently leads to end stage kidney failure [5]. Similarly, transplant-associated TMA is a significant cause of morbidity, mortality, and kidney allograft loss [6,7].

In these disorders, acute microvascular thrombosis of the kidney glomerulus that compromises kidney function is a presenting feature in most cases. *In vitro* studies indicate Stx binds human microvascular endothelial cells (EC) to induce apoptosis [8–12], but a variety of subtle effects on endothelial gene transcription induced by sublethal toxin concentrations may also occur [13,14]. In addition, systemic activation of coagulation proteins both precedes and correlates with subsequent microvascular thrombosis in Shiga toxigenic infection, consistent with a parallel contribution of direct EC injury, and inflammation-driven prothrombotic effects to kidney pathology [15]. Study of the mechanisms of glomerular microvascular injury and repair following Stx administration to rodents, however, are confounded by predominant injury of the kidney tubular epithelial cells *in vivo* in the absence of glomerular EC injury [16,17].

In transplantation, both classical T cell-mediated and antibody-mediated (ABMR) allograft rejection target the microvasculature, recognized in clinical biopsy specimens by features such as subendothelial accumulation of lymphocytes in the allograft arterial intima, glomerulitis, and complement C4d labeling of the endothelium [18]. Endothelial injury in rejection can also be fulminant, typically associated with combined acute cell- and alloantibody-mediated attack, resulting in widespread loss of the endothelium, microvascular thrombosis, and parenchymal cell injury as a consequence of the disturbed microcirculation [19–23]. Isolated allo-antibody-mediated injury has proven difficult to model in rodents [24,25].

We describe a mouse model of acute microvascular endothelial injury selective for kidney glomerular endothelium with synchronized vascular damage and repair. Delivery of a toxin to the glomerular endothelium induces a wave of injury characterized by microvascular thrombosis and fulminant kidney failure. At sublethal doses, glomerular fibrin deposition, microvascular cell apoptosis and EC loss are evident. 

## Materials and Methods

### Animals and ethics statement

Fourteen to 18 week old C57BL/6 female mice (Jackson Laboratory) were maintained according to Canadian Council for Animal Care (CCAC) guidelines under a protocol approved by the Health Sciences Animal Care and Use Committee of the University of Alberta.

### Reagents

The following products were purchased: pure and biotinylated M. *oreades* lectin A (MOA) (EY labs; San Mateo, CA); LPS O55:B5, saporin, and the biotinylated L. esculentum lectin (Sigma-Aldrich; St. Louis, MO); rabbit antibody to cleaved caspase 3 (Cell Signaling Technology; Boston, MA); rat anti-mouse CD31 (BD Pharmingen; Mississuaga, ON); rabbit anti-mouse fibrinogen (GenWay; San Diego, CA); anti-mouse podocalyxin (R&D systems); anti-mouse podocin (Santa Cruz Biotechnology; Santa Cruz, CA); goat anti-rabbit, –rat, or -mouse IgG conjugated to FITC, or DyLight 549 (Jackson ImmunoResearch Laboratories, Inc.). RNA was isolated using RNAeasy Mini Kit (Qiagen; Toronto, ON). The cDNA was prepared using qScript cDNA SuperMix from Quanta Biosciences (Gaithersburg, MD). 

### Conjugation of MOA lectin with saporin

Five mg of pure MOA lectin and saporin were conjugated using sulfo-LC-SPDP as per manufacturer’s instructions (Thermo Scientific, Rockford, IL). The conjugate solution was processed by FPLC and a size-exclusion column (Superdex 75, GE Lifesciences). The resulting fractions of the lectin-saporin conjugate (LS) were analyzed for killing activity against mouse cardiac microvascular endothelial cells (MCEC; [26]); CELLutions Biosystems, Burlington, ON)) *in vitro*. The active fractions of pure LS were pooled, concentrated, and filter-sterilized using an 0.2 micron filter.

### Cleaved caspase 3 assay

MCEC were washed in M199 media, then treated either with 20 μg/mL LS, 20 µg/mL unconjugated lectin, 10 ng/mL TNFα+ 3 μg/mL cycloheximide, PBS, or left untreated and incubated for 4 h at 37 °C and 5 % CO_2_. The cells were fixed and permeabilized, then stained with the anti-cleaved caspase 3 antibody at 1:3000 concentration for one hour. Goat anti-rabbit Dylight 549 secondary antibody was added at a 1:400 dilution. Finally, the caspase 3-positive cells were detected by flow cytometry.

### MOA lectin administration *in vivo*

Five C57Bl/6 mice were injected via tail vein with 10 mg/kg biotinylated MOA lectin. The animals were euthanized by cervical dislocation after 3 h and the following organs were collected: heart, lung, kidney, liver, skeletal muscle, and brain. The organs were placed in OCT solution and snap-frozen in liquid nitrogen. The MOA lectin was visualized using Alexa Fluor 594-conjugated streptavidin (Jackson ImmunoResearch) and confocal microscopy. In subsequent experiments, where indicated, MOA lectin (500 μg/kg), LPS (50 μg/kg), or LS (200 or 500 μg/kg) ± LPS in 100 μL were introduced via retrograde carotid artery injection.

### Histology and immunohistochemical staining

Kidney, heart, lung, liver, spleen, skeletal muscle, and brain were collected and either snap frozen in OCT or placed in IHC zinc fixative (BD Pharmingen). The tissues were stained with Hematoxylin & Eosin (H & E) and with periodic acid- Schiff (PAS). For immunofluorescence staining, 5 μm frozen sections were stained for fibrin, CD31, podocalyxin, podocin, CD42, CD45, or with L. esculentum lectin to specifically label glomerular and peritubular capillary endothelium, and imaged with a spinning disc confocal microscope (Quorum Wave FX-X1). For transmission electron microscopy, tissues ~1 mm^2^ from LPS/ LS or saline injected mice were fixed using Karnovsky fixative solution (Poly Scientific, New York, NY) and embedded in the low viscosity embedding Spurr's Kit (Electron Microscopy Sciences, Hatfield, PA) according to manufacturer's instructions. Ultra-thin sections were stained with uranyl acetate and lead citrate and viewed using a Philips 410 transmission electron microscope.

### Real-time PCR

All PCR primers were designed using Primer Express software (ABI) and produced by Integrated DNA Technologies (San Diego, CA). See Table 1 for a list of the primers used in this study. One μg of total RNA was reversed transcribed into cDNA. SYBR green was used for the real-time PCR (7500 thermocycler, ABI). The data were analyzed using the δδCt relative quantitation method.

**Table 1:** Primers used for qRT-PCR.

Gene	Forward	Reverse
CD31	AGGACGATGCGATGGTGTATAA	AAGACCCGAGCCTGAGGAA
TIE2	GGGCGAAAAAAGTTG TTTGG	CGAACTCGACCTTCACAGAAATAA
eNOS	TGTCTGCGGCGATGTCACTA	CATGCCGCCCTCTGTTG
Nephrin	GCGAGGCACTTCGTGAAAC	CACTTGCTCTCCCAGGAACTCT
KIM1	CCGCAGAAAAACCCTACTAAGG	TGCTCAC AAGGAGCAGTAGCA

### Creatinine analysis

Serial serum samples were sent to the Metabolomics Innovation Center at the University of Alberta to apply a quantitative analysis of creatinine using a combination of direct injection mass spectrometry with reverse-phase LC-MS/MS (Absolute*IDQ* Kit; Biocrates Life Sciences AG, Austria). The serum samples were analyzed with the Absolute*IDQ* kit as directed by the manufacturer.

### Semiquantitative analysis of the renal injury

The histology slides were evaluated by a renal pathologist, DCR, in a blinded fashion. Up to thirty consecutive glomeruli in equatorial section were scored per animal. The kidney sections were scored for glomerular injury, including fibrin and apoptotic cells within the capillary lumen, as a fraction of total glomeruli evaluated as described [27]. Tubular injury was scored using a semi-quantitative scale 0-4 (0- normal, 1- ≤ 25 %, 2- ≤ 50 %, 3- ≤ 75 %, and 4- ≥ 75 % fields affected) as described [28].

### Statistical analysis

The data are expressed as mean ± SD, and analyzed using single or 2-way ANOVA with pairwise comparisons evaluated posthoc using Tukey’s test (GraphPad PRISM, La Jolla, CA). Kaplan-Meier curves were analyzed by the log-rank test. Nonparametric data were analyzed with the Mann-Whitney test. A *p* value < 0.05 was considered significant.

## Results

To generate a potent compound to selectively injure microvascular endothelial cells, we conjugated the toxin, saporin, to the lectin A derived from *M. oreades*. Preliminary testing *in vitro* determined that the lectin-saporin (LS) conjugate, but not saporin or the lectin alone, bound and killed cultured mouse microvascular MCEC, determined by MTT assay of cell viability (data not shown). LS treatment of growth factor-starved MCEC induced apoptosis, indicated by activated caspase 3 staining, of 30.3 ± 5.0% of LS-treated cells versus 0.1 ± 0.1% of PBS-, 1.7 ± 0.4% of lectin-, or 17.5 ± 4.3% Tumor Necrosis Factor-α/cycloheximide-treated control EC (mean ± SEM; n=3 independent experiments; LS *versus* PBS p < 0.05) after 4 hours of treatment. This indicates that the lectin efficiently delivered the toxin to the microvascular EC to induce endothelial apoptosis *in vitro* in the absence of an additional pro-apoptotic stimulus.

**Figure 1:**
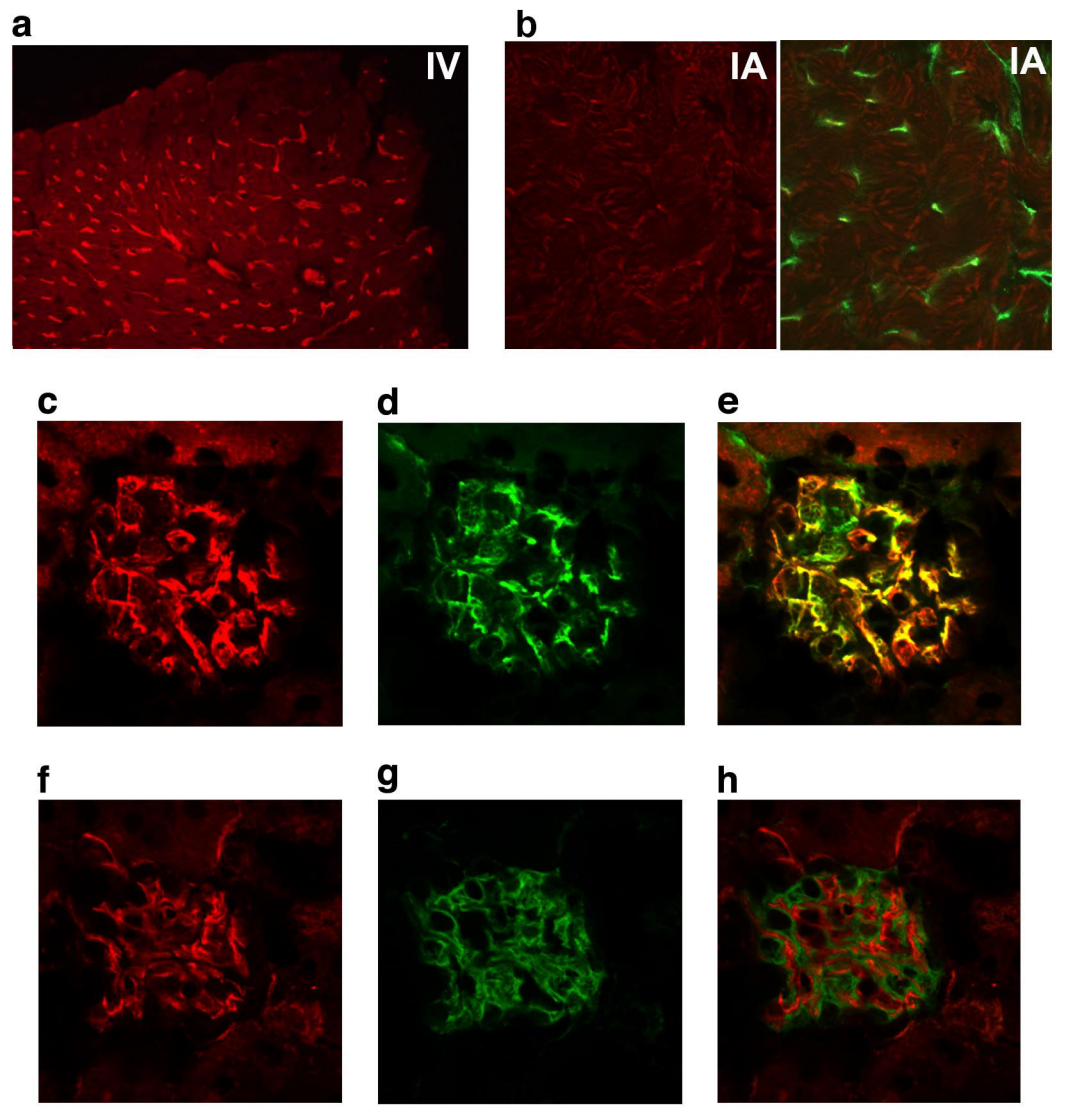
MOA lectin binds the glomerular endothelium. Intravenous injection of MOA lectin labels heart microvascular endothelium (A), but after intra-arterial injection, the kidney glomerular (C, F) but not heart endothelium (B) is labeled. Endothelial cells are labeled with anti-CD31 (green; B (right panel), D), or MOA lectin (red; A, B (left panel), C, F). Double-labeled merged images of CD31 and MOA lectin (E) demonstrate overlapping distribution in the glomerulus. No overlap is identified between MOA lectin (F) and the podocyte marker, podocin (G) in the merged image (H).

Next, we determined the binding characteristics of MOA lectin *in vivo*. Earlier work identifies glomerular microvascular endothelial binding of this lectin [29]. Following *iv* injection into Bl/6 mice, we observed that biotinylated MOA lectin selectively labeled both heart and kidney glomerular microvascular EC (Figure 1). In contrast, when the biotinylated lectin was used as a staining reagent on frozen sections of normal kidney, both glomerular and peritubular capillary EC were uniformly labeled, suggesting that the circulating lectin was largely adsorbed from the blood during transit through the glomerulus. Since we wished to avoid injury to the heart microcirculation, we injected the biotinylated lectin intra-arterially, retrograde via the left carotid artery. We found glomerular but little heart EC labeling using this approach (Figure 1B, C, F). Within the glomerulus we found the lectin selectively bound the EC, but not the mesangial cells or podocytes (Figure 1C-H). We observed no staining of tissue sections from lung, liver, skeletal muscle or brain (data not shown). 

**Figure 2:**
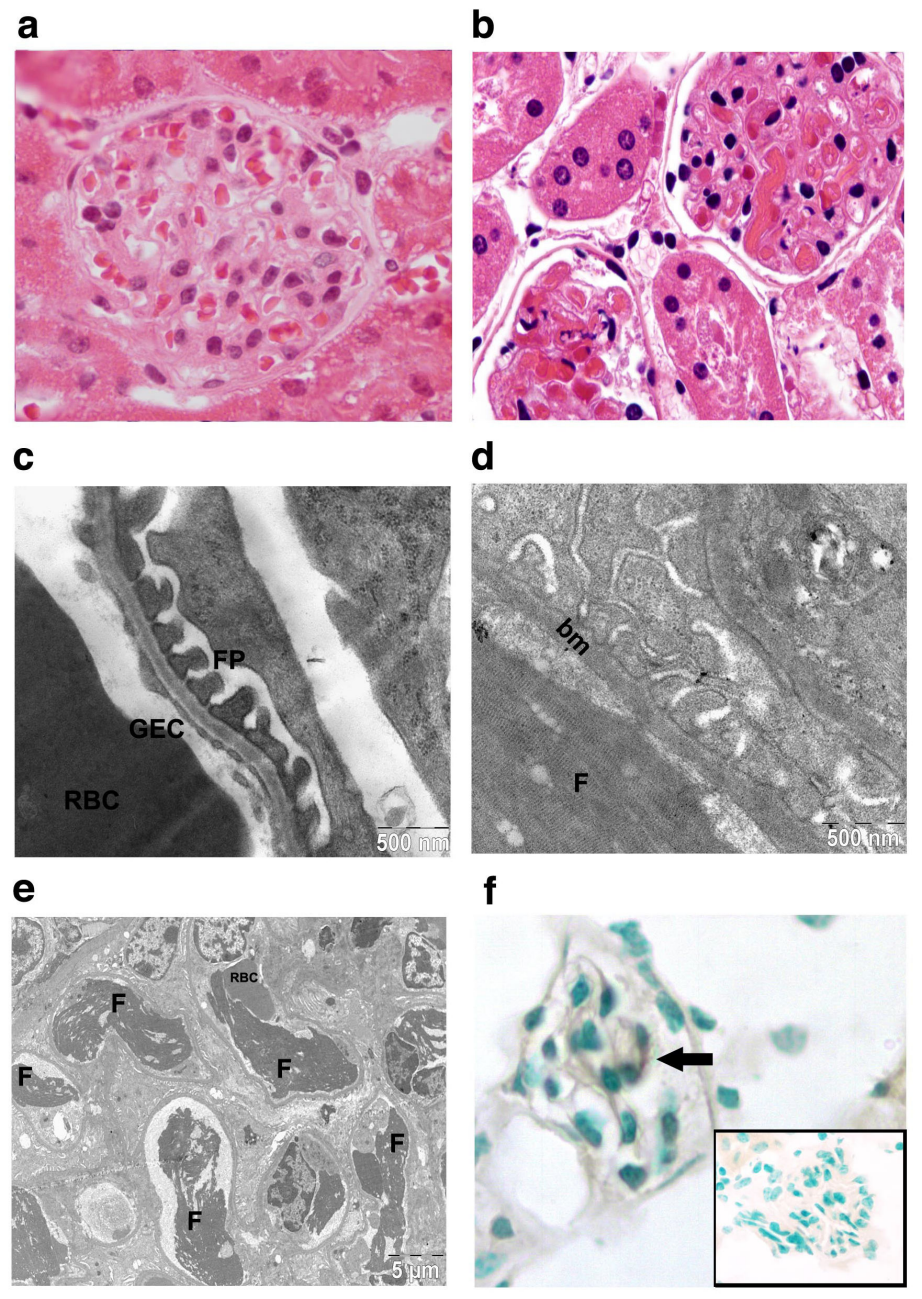
Glomerular thrombotic microangiopathy develops after LPS/ LS treatment. Kidneys were harvested 12 h after saline (A, C) or LPS/ LS (500 μg/kg) treatment (B, D, E, F). Hematoxylin and eosin staining reveals widespread amorphous eosinophilic glomerular capillary thrombi (B), whereas red blood cells are seen in patent capillaries in (A). Transmission electron microscopy shows focal loss of glomerular endothelium (D), and capillary thrombosis (D, E), but preserved podocyte foot processes similar to the control (D *versus* C). Occasional cells in glomerular capillary lumens are found to stain for activated caspase 3 (F; inset: irrelevant antibody control). RBC- red blood cell; GEC- glomerular endothelial cell; FP- podocyte foot process; F- fibrin clot; bm- basement membrane.

**Figure 3:**
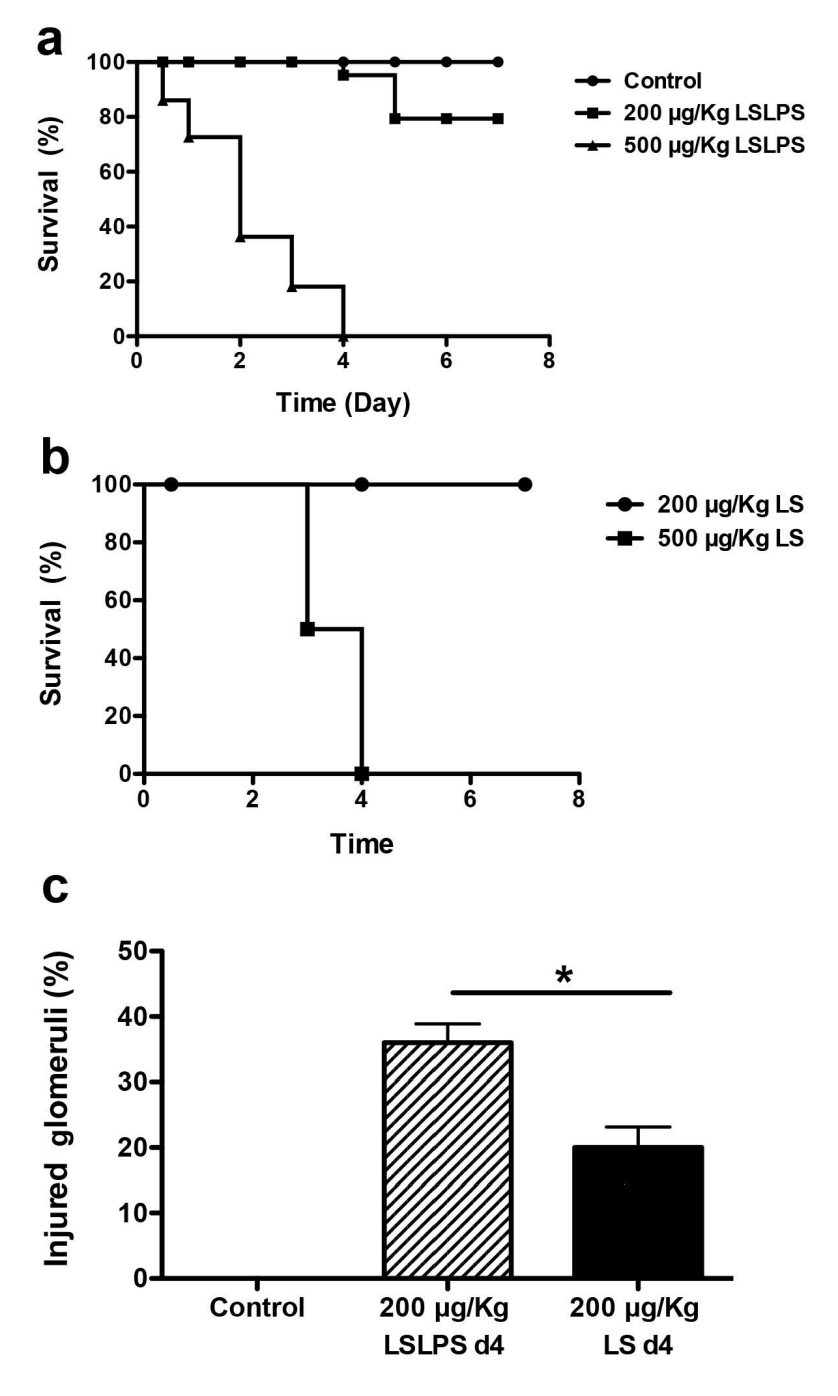
Dose-response effect of LPS/ LS or LS treatment on survival and glomerular injury. Kaplan-Meier survival graph of mice treated with A) saline (n=8), LPS/ LS 500 μg/kg (n=38), or LPS/ LS 200 μg/kg LS (n=24), or B) LS alone 500 μg/kg (n=4), 200 μg/kg LS (n=14). p < 0.001, LS/LPS 500 *versus* LS/LPS 200 μg/kg; p < 0.001, LS 500 *versus* LS 200 μg/kg. c) The fraction of injured glomeruli at Day 4 (n=5 mice/ group; * p < 0.05).

To emulate the pro-coagulant environment associated with many thrombotic microangiopathies, we opted to study mice co-treated with LPS at a threshold dose for tissue factor induction in the kidney [27]. In the first set of experiments, Bl/6 mice were treated with saporin, unconjugated MOA lectin, LS conjugate 500 μg/kg, LPS (50 μg/kg), LPS + LS, or saline, then tissues were harvested at 12 h and examined by a blinded observer (DCR) for evidence of thrombotic microvascular injury. No injury was observed among animals treated with saporin, MOA lectin, or LPS alone. Treatment with the LS conjugate alone, or as shown in Figure 2, treatment with LPS and the LS conjugate, elicited diffuse glomerular capillary thrombosis, but no injury of the heart microvasculature. Glomeruli from three LPS/ LS-injured mice were evaluated for quantitative analysis. We found 72 ± 14% of glomeruli from injured mice showed microvascular thrombosis compared to none from control mice (n=90 glomeruli; p<0.05). Glomerular capillaries stained for fibrin by immunohistochemistry, and fibrin clot was identified in glomerular capillary loops on transmission electron microscopy of LPS/ LS-treated mice (Figure 2C, D, E). The fenestrated EC was absent in many glomerular capillaries examined by electron microscopy (Figure 2D), and luminal cells adjacent to the basement membrane stain for activated caspase 3 by immunohistochemistry (Figure 2F). In contrast, intact podocyte foot process distribution was evident (Figure 2D). Occasional shistocytes could be identified on blood smears. No thrombosis of peritubular capillaries or injury to the tubular epithelial cells was evident at this early time-point. This data demonstrates that LS treatment induces rapid, selective injury of the glomerular endothelium resulting in thrombotic microangiopathy. However, at the 500 µg/kg LS dose, LPS/ LS and LS treatments were uniformly lethal, limiting the utility for experimentation (Figure 3). 

**Figure 4:**
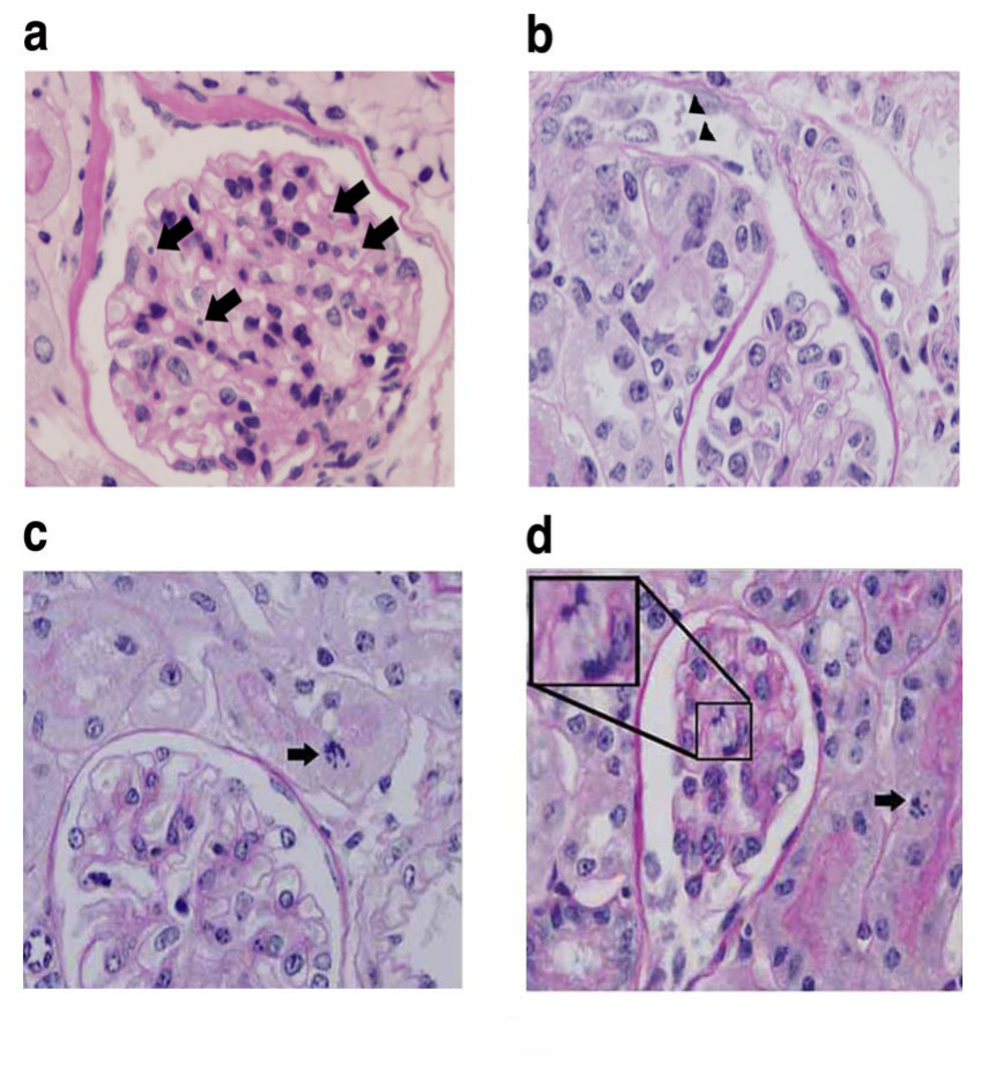
Glomerular microvascular injury after sublethal LPS/ LS treatment. Mice were treated with LPS/ LS 200 μg/kg and tissues were harvested 4 days later and stained with PAS. Apoptotic cells (arrows) are seen in the glomerular capillaries (A) and tubules (B) after LS treatment. Regenerative mitotic changes are evident in the tubular (C) and glomerular capillary compartments (D).

**Figure 5:**
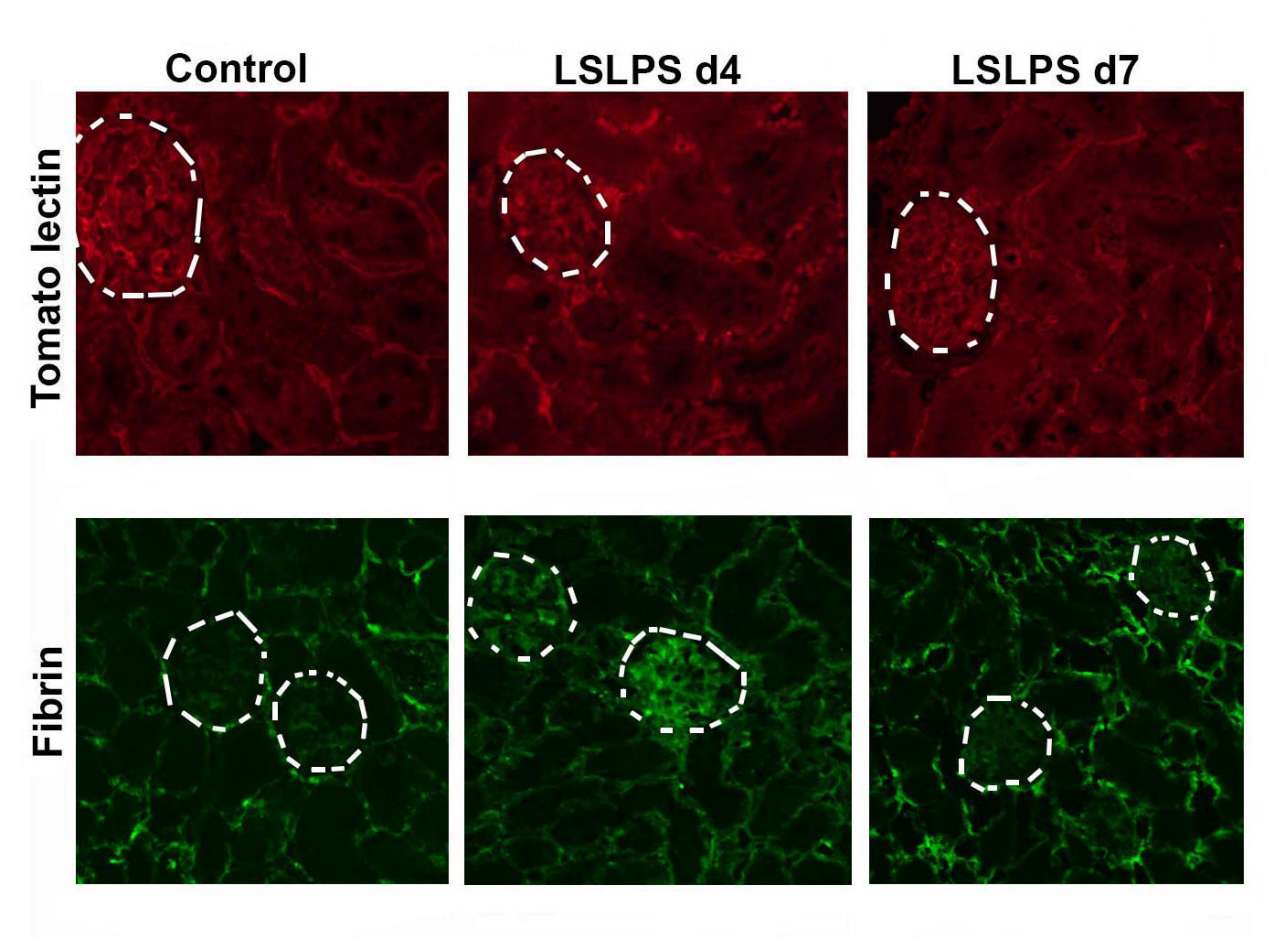
Glomerular microvascular injury after sublethal LPS/ LS treatment. *L. esculentum* lectin labeling of endothelium (upper panels, red) in glomerular (circled) and peritubular capillaries. Glomerular capillary fibrin immunostaining (lower panels, green) after LPS/ LS treatment. Glomerular and peritubular capillary endothelial staining at day 4 appears inhomogeneous, with glomerular fibrin accumulation, and normalizes at day 7. Representative of 5 mice/ group.

In subsequent experiments, mice were treated with 200 μg/kg LS, or LPS/ LS, to characterize the functional consequence of sub-lethal toxin-induced glomerular endothelial injury. This lower dose of LS was tolerated (Figure 3A, B), and glomerular injury was evaluated (Figure 3C). Sub-lethal injury to the microvasculature is reflected at Day 4 by loss of microvascular ECs and regenerative changes most evident in the glomerular endothelial and tubular epithelial cell compartments of the kidney of LPS/ LS-treated mice. As shown in Figure 4, within the glomerulus we identified intralumenal apoptotic cells, frequently positioned adjacent to the capillary wall (Figure 4A). Mononuclear cells were seen in the glomerular capillaries (0.76 ± 0.24 cells/ glomerulus among LPS/LS treated mice *versus* 0.36 ± 0.17 cells/ glo among PBS controls; n=5 mice/ group; p = NS), and PMN were rarely seen. However, only rare CD45-positive leukocytes or CD42-positive platelets were identified in the glomerular microcirculation of LPS/LS-treated or control mice by immunofluorescence staining of frozen sections. Immunostaining for EC was discontinuous in glomerular capillaries of mice treated with the sub-lethal dose of LPS/ LS (Figure 5). Fibrin deposition was evident around the margin of the glomerular capillary loops in immunofluorescence microscopy (Figure 5). In contrast, LS 200 μg/kg given without LPS resulted in no mortality, and little change in glomerular immunostaining for EC or fibrin compared to controls (data not shown).

**Figure 6:**
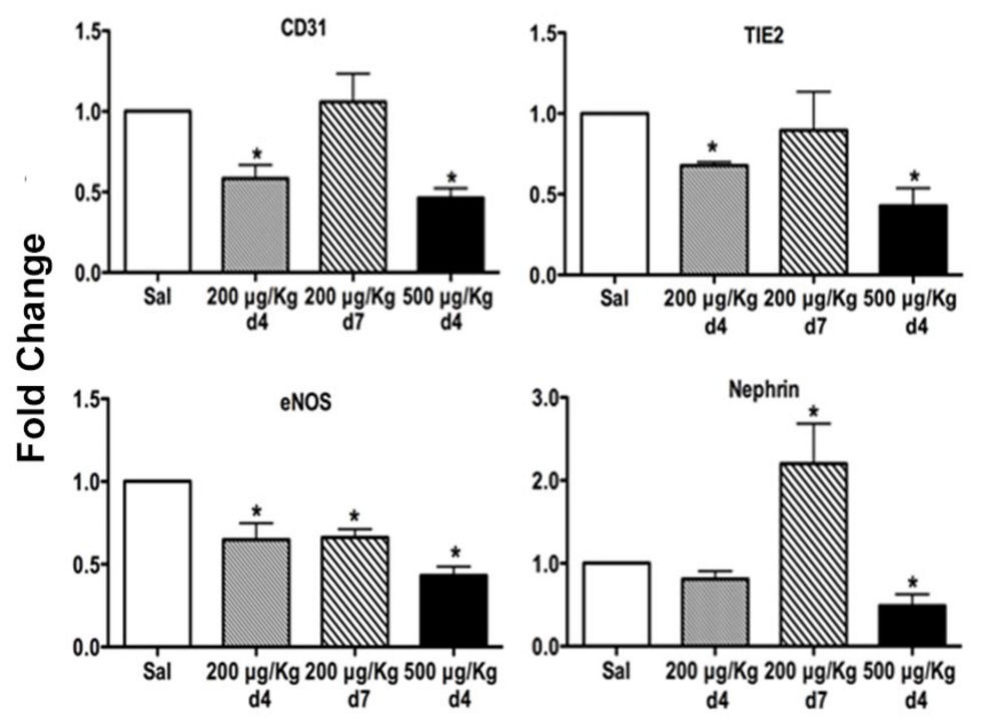
Endothelial gene abundance after glomerular EC injury. Mice were treated with saline or LPS/ LS 200 μg/kg to induce glomerular EC damage, then kidney cortex mRNA was isolated at day 4 or day 7 after injury. Constitutively expressed endothelial genes CD31, TIE2, and eNOS or the podocyte-specific gene nephrin were assessed by qRT-PCR (n = 5 mice/ group; * - p < 0.05 *versus* saline control).

In agreement with these features of glomerular EC injury, the abundance of constitutively expressed EC-specific transcripts, CD31, TIE2, and NOS3, in the kidney cortex was reduced ~40% in sub-lethal LPS/ LS-treated *versus* control mice (Figure 6). Glomerular podocyte foot processes were focally effaced on EM images at Day 4 after injury, but the podocyte-specific transcript nephrin was unchanged during the acute phase of injury between Day 0-4 in LPS/LS-treated animals *versus* controls (Figure 6). 

**Figure 7:**
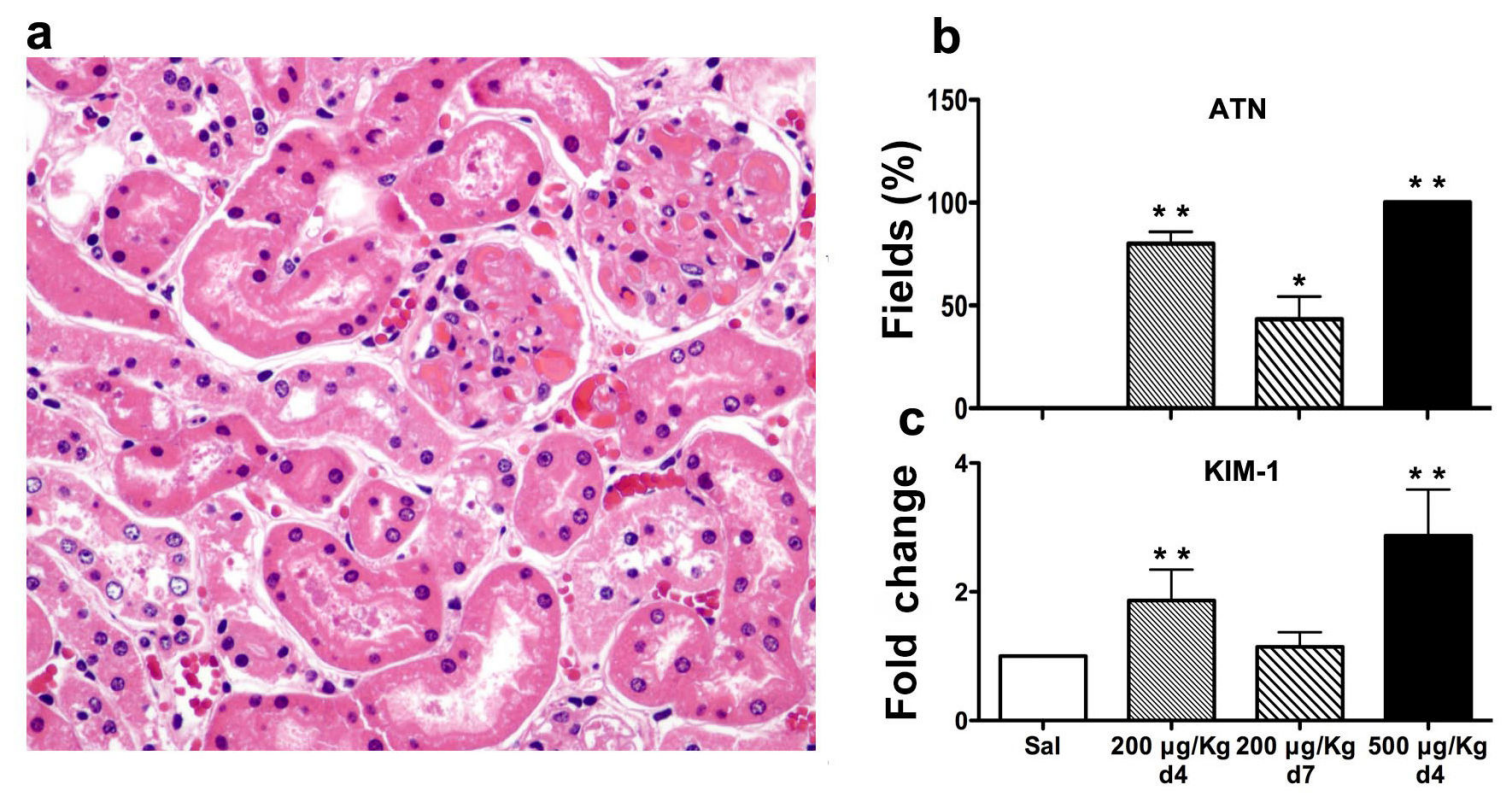
Tubular epithelial cell damage occurs after glomerular EC injury. Tubular epithelial cell effacement and regeneration is evident at day 4 after LPS/ LS 200 μg/kg treatment (A). Features of tubular injury and repair are quantitated in (B) as described in Methods. Expression of the tubular epithelial cell stress gene KIM-1 is quantitated by qRT-PCR in (C). (n = 5 mice/ group; *- p<0.05 *versus* 200 μg/kg day 4 group; **- p<0.05 *versus* saline group).

Injury and regenerative change in the tubular cell compartment of the kidney was prominent at Day 4 as anticipated as a consequence of the disordered microcirculation in the sub-lethally injured animals (Figure 4B, C; Figure 7). Similarly, expression of KIM-1, a tubular epithelial cell stress gene, was elevated in both LS treatment groups at Day 4 after injury. At Day 7 after LPS/LS injury, pathologic features of tubular injury were resolving, and KIM-1 expression normalized (Figure 7). This data indicates that sub-lethal doses of LS induce microvascular endothelial loss that is followed by evidence of tubular epithelial injury and regeneration.

**Figure 8:**
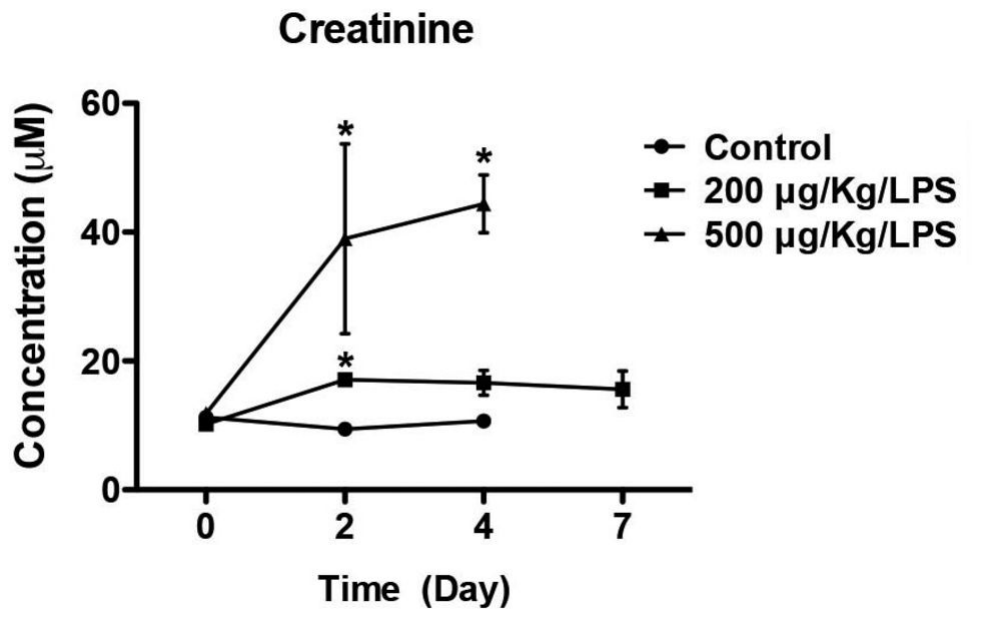
Kidney function is impaired in LS-treated mice. Serum creatinine was assayed by LC/MS/MS. (n = 3-6 mice/ group; p<0.01 LPS/ LS *versus* saline control in 2-way ANOVA. * - p<0.05 *versus* saline control in pairwise comparison).

Repair of the injured vascular endothelium was evident on Day 4 and Day 7 tissue sections in the sublethal LS injured group, reflected by mitotic figures within the glomerular capillaries (Figure 4C, D), normalization of glomerular and peritubular capillary immunostaining for EC and fibrin (Figure 5), and endothelial-specific gene expression (Figure 6) at Day 7. Serum creatinine, as a measure of kidney function, was increased in the lethal LPS/LS dose group, consistent with acute renal failure associated with glomerular TMA injury. The creatinine showed an intermediate rise in the LS 200 μg/kg dose groups *versus* baseline, and was normalizing by Day 7 (Figure 8).

## Discussion

Endothelial injury of the kidney glomerular microvasculature is a key feature of diverse diseases resulting in thrombotic microangiopathy. The consequences of these disorders on organ function represent a considerable health burden to affected individuals. In particular, Stx-mediated glomerular EC injury in epidemic toxigenic *E coli* infections contributes to morbidity and mortality of both children and adults [1–4]. Moreover, among transplant recipients, cell-mediated allo-immune responses, and the more recently recognized antibody-mediated rejection, target the EC in kidney allografts [20]. Although low-grade injury is tolerated for a time, the cumulative burden of damage to the microvasculature ultimately limits kidney allograft survival [30–33]. Animal models of this disease are needed to develop an understanding of the mechanisms of endothelial defense, consequences of injury, and key repair mechanisms that might be exploited to minimize organ damage or prolong graft survival.

We describe a new model of temporally coordinated glomerular EC damage in the mouse, and characterize the immediate consequences of glomerular microvascular compromise on the kidney. With marked injury, widespread thrombosis of the glomerular capillaries is seen, followed by features of tubular injury and functional compromise of the kidney precipitating death in a few days. These features resemble the early phase of microvascular injury observed in clinical specimens with Stx- or alloantibody-mediated damage [20,21,23,34–37]. More limited endothelial injury, on the other hand, provokes moderate functional compromise associated with features of repair in the vascular and tubular cell compartments. 

Other approaches to model thrombotic microangiopathy in the mouse have been developed. Mutations in complement regulatory proteins [38], or vascular endothelial growth factor [39] elicits asynchronous, chronic progressive microvascular thrombosis and ultimately death. Administration of Shiga-like toxins elicit acute kidney injury in the mouse [14,40], and directly damages kidney tubular epithelial, but not glomerular endothelial cells [16,17]. In a model of allo-immune EC injury, transplantation into CCR5-deficient mice sensitized to donor allo-antigen, elicits microvascular complement deposition and heart allograft rejection as a consequence of antibody binding to allogeneic EC and cardiac myocytes [24,25]. Selective injury of the microvasculature has been approached using lectins that label the endothelial glycocalyx after intravenous injection [41,42]. Concanavalin A, for example, binds endothelium from diverse microvascular beds in rodents, but selective injection into a renal artery followed by anti-concanavalin A antibody induces widespread microvascular injury associated with inflammatory changes in the glomerulus and peritubular capillaries [43]. 

These valuable models support investigations of the role of complement, alloantibody, and innate immune cell actions on endothelium, but often induce asynchronous complex injury that may not be selective for the kidney microvascular endothelium. Deficient expression of one or more components of the complement system in many inbred laboratory mouse strains may confound these approaches [44]. The current model has the strength of widespread, synchronized, and selective GEC injury resulting in reproducible TMA and compromised kidney function. The approach is less technically challenging than the concanavalin A model, since selective renal artery cannulation is not required. Like the Stx A subunit, the saporin moiety of the LS conjugate is known to inhibit ribosome-dependent translation, hence this injury model closely emulates the mechanism of the human hemolytic uremic disease, but lacks the confounding effect of direct Stx toxicity to tubular epithelial cells [16,17,45].

The MOA lectin has been characterized to specifically bind to the Gal-α(1,3)Gal epitope on glycoproteins displayed on the glycocalyx of mouse glomerular endothelium [29]. Injection of the MOA lectin alone at high doses was reported to elicit proteinuria, but not features of diffuse TMA, consistent with our observations. In contrast to this previous report, we observed the lectin binding to the heart microvascular EC *in vitro* and *in vivo* in Bl/6 mice after *iv* injection, but selective kidney targeting could be achieved by systemic intra-arterial injection. Injury of the heart microvasculature is under study to model the effects of microvascular injury and repair in that organ.

Injury of the glomerular endothelium was evident in both the high- and low-dose LS treatment groups. At early timepoints, we observed focal loss of the glomerular endothelium and EC injury reflected by loss of fenestration on transmission electron microscopy, inhomogeneous glomerular EC immunostaining, and focal fibrin deposition in glomerular capillaries after LPS/ LS treatment. This morphological data is supported by decreased transcript abundance of the characteristic constitutively-expressed endothelial genes CD31, TIE2, and NOS3. In contrast, injury to the podocyte, tubular cell, and peritubular capillary compartments is evident after the GEC injury. Taken together this data indicates primary GEC injury induced by LPS/ LS treatment, resulting in features of TMA.

The addition of low-dose LPS to the LS toxin conjugate enhanced the reproducibility of glomerular injury. A low dose challenge with LPS was used to promote a prothrombotic environment [27,40,46], and we observed no effect of LPS alone on the kidney histology or function, consistent with previous reports [17,40]. Although LPS alone has been used as a model of kidney injury in lethal septicemia, the dose of LPS administered here is ~100 fold lower. Nevertheless, LPS in combination with cycloheximide, a ribosome inhibitory toxin similar in action to saporin, is known to induce EC apoptosis *in vitro* [47,48], hence we also expect that EC injury in LS-treated animals was enhanced by LPS. Together these agents reproduce the two important components, EC injury and hypercoagulability, of the pathophysiology thought to be involved in the development of human thrombotic microangiopathy [15].

In summary, we describe a mouse model of selective glomerular endothelial injury that elicits the pathological features of TMA similar to those seen in Stx-mediated acute kidney injury, and antibody-mediated allograft rejection. The synchronized endothelial injury is associated with kidney dysfunction and should facilitate investigation of mechanisms underlying defense and repair of the microvasculature, and recovery of organ function.
